# The effect of radio-adaptive doses on HT29 and GM637 cells

**DOI:** 10.1186/1748-717X-3-12

**Published:** 2008-04-23

**Authors:** Silke B Schwarz, Pamela M Schaffer, Ulrike Kulka, Birgit Ertl-Wagner, Roswitha Hell, Moshe Schaffer

**Affiliations:** 1 Department of Radiation Oncology, Ludwig-Maximilians-University Munich, Marchioninistr. 15, 81377 Munich, Germany; 2 Institute of Clinical Radiology, Ludwig-Maximilians-University Munich, Marchioninistr. 15, 81377 Munich, Germany

## Abstract

**Background:**

The shape of the dose-response curve at low doses differs from the linear quadratic model. The effect of a radio-adaptive response is the centre of many studies and well known inspite that the clinical applications are still rarely considered.

**Methods:**

We studied the effect of a low-dose pre-irradiation (0.03 Gy – 0.1 Gy) alone or followed by a 2.0 Gy challenging dose 4 h later on the survival of the HT29 cell line (human colorectal cancer cells) and on the GM637 cell line (human fibroblasts).

**Results:**

0.03 Gy given alone did not have a significant effect on both cell lines, the other low doses alone significantly reduced the cell survival. Applied 4 h before the 2.0 Gy fraction, 0.03 Gy led to a significant induced radioresistance in GM637 cells, but not in HT29 cells, and 0.05 Gy led to a significant hyperradiosensitivity in HT29 cells, but not in GM637 cells.

**Conclusion:**

A pre-irradiation with 0.03 Gy can protect normal fibroblasts, but not colorectal cancer cells, from damage induced by an irradiation of 2.0 Gy and the application of 0.05 Gy prior to the 2.0 Gy fraction can enhance the cell killing of colorectal cancer cells while not additionally damaging normal fibroblasts. If these findings prove to be true in vivo as well this may optimize the balance between local tumour control and injury to normal tissue in modern radiotherapy.

## Background

It is widely accepted that the shape of the dose-response curve at low doses differs from the linear quadratic model [1]. Induced radioresistance, hyperradiosensitivity or adaptive responses (i.e. a biopositive effect induced by a low priming dose and identified after application of a higher challenging dose) may occur at low doses of irradiation. The radio-adaptive response was first recognized 1984, when Olivieri et al. demonstrated that human lymphocytes exposed to low concentrations of radioactive thymidine show fewer chromatid aberrations caused by a 1.5 Gy challenging dose than those not pre-exposed to irradiation [2]. Several publications have studied the effect with different cell lines, different pre-irradiation doses, and variable challenging doses [3-10]. However, the exact mechanism of the effect is yet unknown, thus precluding predictions whether a cell line will show an adaptive response or not. An altered gene expression caused by low-dose ionizing radiation has been identified. A radio-adaptive response seems to be associated with an up-regulation of DNA repair and stress response genes and a down-regulation of cell cycle control and apoptosis genes. TP53 (Tumour Protein p53) is supposed to play an important role in this mechanism [11]. Protein synthesis, metabolism and signal transduction appear to be involved in the adaptive response as well [9]. However, controversy remains regarding the mechanism and role of the adaptive response [12]. This is probably due to cell-type and tissue-specific variations and different experimental conditions [12,13].

Most radio-adaptive response experiments focussed on basic research of this phenomenon, with only few studies concentrating on its clinical applications, e.g. in radiotherapy [14].

We had previously described a difference between the reaction of normal bladder cells (HCV29) and that of bladder cancer cells (RT4) to different adaptive doses of irradiation. HCV29 cells showed an induced radioresistance after pre-irradiation doses of 0.05 Gy or 0.1 Gy, whereas RT4 cells displayed a hyperradiosensitivity after pre-irradiation with 0.05 Gy, 0.1 Gy, 0.2 Gy or 0.5 Gy [14].

While bladder cancer is only infrequently treated by radiotherapy, pre- or postoperative irradiation of stage II or III colorectal cancer is very common. These tumours are responsible for 655.000 deaths/year worldwide [15] with an incidence of 88.3/100.000 men and of 84.9/100.000 women in Germany in 2002 [16]. Colorectal cancer is thus one of the most common cancers after prostate cancer for men and breast cancer for women. It is therefore of utmost importance to optimize the treatment for colorectal cancer in order to attain a high cure rate and minimized side effects. Radio-adaptive doses applied may probably aid to achieve this end as an adjunct to standard chemo-radiotherapy.

It was therefore our aim to evaluate the effect of different pre-irradiation doses followed by a 2.0 Gy fraction on a colorectal cancer cell line (HT29) and on normal fibroblasts (GM637).

## Methods

### Cell culture

HT29 is a cell line derived from human colorectal cancer cells [17], while GM637 is a cell line of human fibroblasts [18].

Both cell lines were routinely grown in 80 ml flasks (NUNC, Wiesbaden, Germany). For HT29 cells the medium consisted of 83% McCoy's 5A medium supplemented with 16% fetal calf serum and 1% of a mixture of antibiotics (10^4 ^IU penicilline/ml and 10^4 ^μg streptomycin/ml). The medium for GM637 cells was a mixture of 82% minimum essential medium MEM (Eagle) with Earle's salts, 25 mM HEPES and without L-glutamine, of 16% fetal calf serum, of 1% sodium pyruvate 100 mM and of 1% of the antibiotic mixture (10^4 ^IU penicilline/ml and 10^4 ^μg streptomycin/ml). The cell lines were incubated at 37°C with 5% CO_2_, 95% humidity and a pH of 7.4. Cells were passaged in the exponential growing phase once a week, using 0.05% trypsin plus 0.02% EDTA in PBS at 37°C.

### Experimental plating

96-well culture plates were used for all experiments. Cells were seeded at a density of 250 cells per well (250 cells in 200 μl medium). Each plate contained wells with HT29 and wells with GM637, so that both cell lines were treated in the exact same way. Additionally, another plate was seeded with an increasing cell number per row (62.5-125-250-500-750 cells per well) for cell growth monitoring and survival reference.

### Irradiation

After an incubation period of 24 h the plates were irradiated with 0 Gy, 0.03 Gy, 0.05 Gy or 0.1 Gy at a dose rate of 0.03 Gy/min (225 kV, 5 mA, 0.35 mm Cu). 4 h after pre-irradiation cells were further irradiated with 0 Gy or 2.0 Gy at a dose rate of 1.0 Gy/min (225 kV, 15 mA, 0.35 mm Cu). As a result, eight different irradiation groups were evaluated: 0 Gy (control), 0.03 Gy alone, 0.05 Gy alone, 0.1 Gy alone, 2.0 Gy alone, 0.03 Gy plus 2.0 Gy, 0.05 Gy plus 2.0 Gy and 0.1 Gy plus 2.0 Gy.

We chose the pre-irradiation doses to be 0.03 Gy, 0.05 Gy and 0.1 Gy respectively following an earlier study [14] that demonstrated pre-irradiation doses of 0.05 Gy and 0.1 Gy, but not of 0.5 Gy to be effective.

### Cell viability test

The plates were incubated for an additional 7 days. The medium was subsequently removed from all wells. Cells were washed with PBS and 100 μl medium with 10% WST-1 (tetrazolium salt 4- [3-(4-iodophenyl)-2-(4-nitrophenyl)-2H-5-tetrazolio]-1,3-benzene disulfonate) were added to all wells. WST-1 is cleaved to a water-soluble formazan dye whose amount directly correlates to the number of metabolically active cells and is quantified spectrophotometrically by an ELISA reader at a wavelength of 450 nm (reference wavelength: 690 nm). The optical density was measured immediately (background measurement) and after 3 h.

### Result analysis

All experiments were repeated three times resulting in at least 55 single data sets per irradiation group and cell line. The standard curve for control cells was checked to be certain that the cells of the experimental plates are in the exponential phase of the survival curve and not in the plateau phase. After subtracting the background the relative cell survival of all wells was calculated. Using the Student's t-test the statistical significance of the results (p ≤ 0.05) was evaluated.

## Results

### HT29 cell studies

An irradiation with 0.05 Gy (p = 0.000002) and 0.1 Gy (p = 0.000136) led to a significantly lower cell survival in HT29 cells, whereas HT29 cells irradiated with 0.03 Gy did not show a significant decrease in cell survival, when compared to the control group (Table 1).

**Table 1:** Descriptive statistical parameters of the experiments on the effect of different low irradiation doses alone on HT29 cells

Irradiation dose	Mean survival	Standard deviation	p-Value
0 Gy	0.784	0.148	-
0.03 Gy	0.736	0.101	0.367357
0.05 Gy	0.508	0.052	0.000002*
0.1 Gy	0.604	0.036	0.000136*

* statistically significant

The adaptive response experiments, i.e. the experiments performed with a pre-irradiation followed by a 2.0 Gy irradiation, did not demonstrate a significant induced radioresistance. The 0.05 Gy pre-irradiation dose even led to a significantly decreased cell survival (p = 0.012249). (Table 2).

**Table 2:** Descriptive statistical parameters of the experiments on the effect of different pre-irradiation doses plus 2.0 Gy on HT29 cells

Irradiation dose	Mean survival	Standard deviation	p-Value
2.0 Gy	0.501	0.023	-
0.03 Gy + 2.0 Gy	0.551	0.044	0.066972
0.05 Gy + 2.0 Gy	0.434	0.025	0.012249*
0.1 Gy + 2.0 Gy	0.527	0.033	0.288153

* statistically significant

### GM637 cell studies

An irradiation dose of 0.03 Gy (p = 0.711896) alone did not result in a significantly lower cell survival of GM637 cells, while irradiation doses of 0.05 Gy (p = 0.000003) and 0.1 Gy (p = 0.008301) led to a significantly reduced cell survival (Table 3).

**Table 3:** Descriptive statistical parameters of the experiments on the effect of different low irradiation doses alone on GM637 cells

Irradiation dose	Mean survival	Standard deviation	p-Value
0 Gy	0.785	0.116	-
0.03 Gy	0.804	0.123	0.711896
0.05 Gy	0.539	0.053	0.000003*
0.1 Gy	0.659	0.084	0.008301*

* statistically significant

Pre-irradiation doses of 0.03 Gy (p = 0.002591) or 0.1 Gy (p = 0.044575) applied 4 h prior to the 2.0 Gy fraction led to a significantly enhanced cell survival in GM637 cells, when compared to cells irradiated with 2.0 Gy alone. These pre-irradiation doses therefore led to an induced radioresistance in GM637 cells. This effect was most pronounced in the 0.03 Gy experiment. A pre-irradiation of 0.05 Gy led to a slightly increased radioresistance, which was not statistically significant however (p = 0.429477). (Table 4).

**Table 4:** Descriptive statistical parameters of the experiments on the effect of different pre-irradiation doses plus 2.0 Gy on GM637 cells

Irradiation dose	Mean survival	Standard deviation	p-Value
2.0 Gy	0.421	0.027	-
0.03 Gy + 2.0 Gy	0.516	0.049	0.002591*
0.05 Gy + 2.0 Gy	0.449	0.057	0.429477
0.1 Gy + 2.0 Gy	0.479	0.037	0.044575*

* statistically significant

### HT29 and GM637 cell studies in comparison

An irradiation with 0.03 Gy alone did not have a significant effect on the survival of HT29 and GM637 cells, whereas 0.05 Gy and 0.1 Gy led to a significantly lower cell survival in both cell lines.

The effect of the various pre-irradiation doses applied 4 h prior to the 2.0 Gy fraction varied between HT29 and GM637 cells. Pre-irradiation doses of 0.03 Gy and 0.1 Gy induced a significant radioprotective effect in GM637 fibroblasts, but not in HT29 colorectal carcinoma cells. A pre-irradiation dose of 0.05 Gy led to a significantly lower cell survival in HT29 cells, and a slightly, not significantly, higher survival in GM637 cells. A pre-irradiation with 0.03 Gy seems to therefore protect normal fibroblasts, but not colorectal cancer cells, from radiation-induced damage, while an adaptive dose of 0.05 Gy can lead to a reduced survival of colorectal cancer cells, but not of normal fibroblasts.

## Discussion

Modern radiotherapy uses sophisticated techniques to optimize therapeutic tumour control. Side effects on normal tissues, however, are the single most limiting factor to the therapy. Therefore research in the field of radiation oncology not only needs to focus on maximizing tumour destruction but also on minimizing side effects on normal tissues.

The results of our studies imply that a low-dose pre-irradiation applied 4 h prior to the main irradiation may either cause a reduction of the side effects of radiotherapy of colorectal carcinomas on normal tissues or allow enhanced tumour cell killing while not leading to additional side effects – provided that our findings prove to be true in vivo as well.

In our experiments, 0.03 Gy by itself did not have a significant effect on cell survival, neither on the tumour (HT29) nor on the normal cell line (GM637). When this dose is applied as a pre-irradiation dose it may induce a significant radioprotective effect in GM637 human fibroblasts, but not in HT29 colorectal cancer cells. Provided that this phenomenon not only exists in vitro, but also in vivo, and that our cell model reflects real tissue conditions, and moreover exerts its effect for several dose fractions, reduced side effects may be achieved for radiotherapy of colorectal cancer. Using an adaptive dose to protect normal tissue may allow a dose escalation to result in a destruction of more tumour cells. In that case, a better downstaging may theoretically be achieved thus allowing more radical resections. The addition of a daily dose of 0.03 Gy to the conventional 1.8 Gy or 2.0 Gy fractions would add no more than 0.9 Gy to the total irradiation dose applied in approx. 30 sessions.

Hints for an alternative possible application of pre-irradiation doses in radiotherapy of colorectal cancer might result from our experiments, as well. A pre-irradiation dose of 0.05 Gy led to a significantly lower cell survival in HT29 cells, and a slightly, not significantly, higher survival in GM637 cells. When an adaptive dose of 0.05 Gy can lead to a reduced survival of colorectal cancer cells, but not of normal fibroblasts, the pre-irradiation can help to improve tumour cell killing in cancer therapy while not adding more side effects.

Clinical studies are, however, needed to evaluate whether this assumptions holds true in a clinical setting.

In our study, we have concentrated on the commonly known doses for radio-adaptive response experiments. We have therefore not used a pre-irradiation dose of less than 0.03 Gy. As we demonstrated the pre-irradiation dose of 0.03 Gy to be effective to induce radioresistance in normal fibroblasts, it remains to be investigated, however, whether a dose below 0.03 Gy may also lead to the above mentioned effects.

Lambin et al. demonstrated HT29 cells to be hypersensitive to low radiation doses. While the cell survival response showed a good fit to the linear quadratic model for 2 to 5 Gy, it demonstrated a hyperradiosensitivity for 0.05-0.3 Gy and an induced radioresistance for 0.3-1.0 Gy [19]. This is consistent with our findings that HT29 cells show a significantly lower survival when irradiated with 0.05 Gy or 0.1 Gy alone. A dose of 0.03 Gy was not tested by Lambin et al.. In our experiments 0.03 Gy alone did not have a significant effect on the survival of HT29 cells.

Cell lines known to be relatively radioresistant, e.g. HT29 cells (colorectal cancer) and RT4 cells (bladder cancer), often demonstrate a hyperradiosensitive reaction to low irradiation doses [14,20,21]. It remains to be clarified, whether this hyperradiosensitivity is an independent effect or whether it represents the absence of induced radioresistance [20]. The induction of PBP74/mortalin/Grp75, a member of the hsp 70 family, seems to play a role in induced radioresistance in HT29 cells [6].

A radio-adaptive response can be measured in terms of cell survival – as performed in our study -, of reduction of chromosomal aberrations, of micronuclei formation or of mutations [3,22-24]. It occurs after pre-irradiation doses of 0.01 Gy [4] to 1.5 Gy [3] depending on the cell line examined and on the experimental conditions. In our study, we observed a radio-adaptive response in GM637 cells for 0.03 Gy and 0.1 Gy pre-irradiation doses. The time span of 4 h between pre-irradiation and the challenging dose has been used in the past [25], however, other intervals have been studied as well with differing results [26].

The mechanism of the adaptive response is still not completely understood, but it is widely accepted that inducible DNA repair mechanisms play an important role [27], whereas others believe in decreased damage fixation [28]. Furthermore, stress response, apoptosis pathways, signal cascades, DNA conformation changes, chromosome organization, bystander effects and cell cycle control are probably involved as well [1,29-33]. Protein synthesis appears to be essential for the induction of an adaptive response [5] and several genes have been identified that play a crucial role in this phenomenon [6-10]. Recent publications pointed out the role of the MAPKs p38 and ERK1/2 [34], NF-κB [35] and activation of Raf and Akt [36]. It is proposed that the radio-adaptive response follows mainly from mutations at the base-sequence level, not the chromosome level, [37] and involves some components of the nucleotide excision repair pathway [38]. Adaptive and bystander response are presumably linked via reactive oxygen and nitrogen species [39].

Further experiments will be needed to completely elucidate the mechanism of the adaptive response. In addition, potential clinical applications of the adaptive response need to be studied as well. Our data and previous studies suggest normal and tumour cells to react differently to low pre-irradiation doses. While bladder cancer cells show a hyperradiosensitivity, normal bladder cells demonstrate an induced radioresistance [14].

## Conclusion

In conclusion, we demonstrated a pre-irradiation with 0.03 Gy to protect human fibroblasts (GM637) but not colorectal cancer cells (HT29) from radiation induced damage of a subsequent 2.0 Gy challenging dose and the application of 0.05 Gy prior to the 2.0 Gy fraction to enhance the cell killing of colorectal cancer cells while not additionally damaging normal fibroblasts. If these findings prove to be true in vivo as well this confirms the hypothesis that low pre-irradiation doses may optimize the balance between local tumour control and injury to normal tissue in modern radiotherapy of colorectal cancer, one of the most common neoplasms world-wide.

## Competing interests

The authors declare that they have no competing interests.

## Authors' contributions

SBS: designed protocol, conducted data evaluation, wrote the article

PMS: designed protocol, conducted data evaluation, wrote the article

UK: collected data, statistical analysis, laboratory controlling

BEW: statistical analysis, critical review of the manuscript

RH: biological technical assistant

MS: designed protocol, conducted data evaluation, critical review of the manuscript, group supervisor

All authors read and approved the final manuscript.
